# Sulphate of Cinchonidia

**Published:** 1875-09

**Authors:** Edmund Andrews

**Affiliations:** Professor of Surgery in Chicago Medical College, etc.; Chicago


### SULPHATE OF CINCHONIDIA.

By EDMUND ANDREWS, M.D.,

Professor of Surgery in Chicago Medical College, etc.

This is one of the neglected products of Peruvian bark, which is, apparently, worthy of a much more extensive use than it has hitherto had, especially as it is nearly equal to quinine in efficiency, is less disposed to disturb the brain, and is far cheaper.

The following facts and statements are of interest in this connection. My own experience with it is not large, because a surgical practice affords few opportunities to test it as an antiperiodic; but the results of observations made by others have fallen into my hands in such a manner as to seem worthy of decided attention.

*Case First* was attacked with a headache, which, by previous experience was expected to last one or two days. Nine grains of sulphate of cinchonidia, taken at one dose, stopped the pain in half an hour, as a similar dose of quinine had occasionally done before in the same patient.

*Case Second* was a lady, laboring under a slow debility from various causes, and who had been in the habit of using preparations of quinine, etc., as a tonic. This lady was very sensitive to such remedies, and readily recognized their impression upon her. She stated that the cinchonidia acted on her exactly like quinine, except that it produced no unpleasant sensation in the head.

*Case Third* was a neuralgia, which yielded to a large dose of cinchonidia, very much as it probably would have done to quinine.

Prof. Lester Curtiss, of Chicago, gives me the following observations: "I have prescribed it in dispensary practice, in malarial troubles of children, in about the usual doses that I have been accustomed to use of quinine, with the result, usually, of interrupting the symptoms. The cases in which I have employed it, however, were mild attacks. I have found it of use also, in one or two instances, in mitigating the symptoms of hectic occurring in consumption.

"I have tried the drug on myself also. Feeling the premonitory symptoms of intermittent, I began taking the cinchonidia in indefinite quantities, and at irregular intervals. I probably took from two to four grains two or three times a day, for a week or more, occasionally missing a day. The cinchonidia was not sufficient to prevent the attack, and last Wednesday, about nine or ten o'clock in the evening, I had a febrile attack, without any chill preceding. The next day I took four doses of the cinchonidia, of about five grains each; the febrile attack returned the following evening, a little earlier than before. The next day I took two doses of about eight grains each, in connection with five grains of quinine; the fever returned again in the evening, with more severity than before. The next day, Friday, I took fifteen grains more with five grains of quinine, and the attack was still more severe than the day before. My measurement of the cinchonidia was approximate, but I took the trouble to go to a druggist and have him weigh out five grains, so that I could estimate the quantity more correctly, and I think that my estimate statements of quantity are tolerably correct."

Prof. D. T. Nelson, of Chicago, informed me that he had used the sulphate of cinchonidia in Mercy Hospital several times, in the treatment of intermittent fever, generally with good success. In one case, however, of intermittent, complicating Bright's disease of the kidneys, he had failed to control the intermittent with the cinchonidia, but he also found that it was only with difficulty that quinine controlled the trouble. He also stated that he found the cinchonidia less apt to affect the stomach than quinine, and that he had not met with any of the head symptoms which so commonly result from the administration of the latter drug.

Prof. Hollister, of Chicago, gave the cinchonidia a short trial during his service in Mercy Hospital, last September. He does not remember the particulars of his trial, which was not very thorough or systematic. His general impression of the results of his trial is, that it was unsatisfactory, and that the drug would only be of use in the class of cases in which salicin would have the advantage.

I find, quoted elsewhere, the following opinions of gentlemen personally known to me, viz.:

Dr. Wm. E. Quine, Professor Materia Medica, etc., Chicago Medical College, Chicago, April, 1875:—"So far as my experience in the use of cinchonidia has gone, it certainly justifies me in the belief that it ranks equal in power as a tonic and antiperiodic with any of the other derivations of the cinchona."

Prof. H. M. Lyman, Physician County Hospital, Chicago, Ill., May 3, 1875:—"I am now trying the sulphate of cinchonidia with much satisfaction at the County Hospital."

Dr. A. R. Bodley, Athens, Calhoun Co., Mich., November 16, 1874:—"I have given the sulphate of cinchonidia a very fair test, and pronounce it fully equal to sulphate of quinia in malarious diseases. I have used it in about forty cases of intermittent and remittent, myself among the number, and in no case have I failed to produce the desired effects. I thought when I first examined it, that its insolubility in alcohol, acids, etc., would render it objectionable, but I find it produces little or no cerebral disturbance, even when given in large doses. I am satisfied the disease is not as likely to recur after being broken, as when the sulphate of quinia is used.

"I combine about five grains of capsicum to the ounce, and I think it adds to its efficiency, as it does also with quinia."

Statements are published that extensive statistical experiments were made in India, with the various alkaloids of cinchona, which showed that cinchonidia seldom failed to arrest intermittents, ranking, in that respect, so close to quinia as to be practically equal.

Owing to my own practice being purely surgical, I only see intermittents when they occur as a complication in surgical cases, hence I am unable to present any statistics of my own, but the conclusion impressed on my mind is, that cinchonidia is an extremely valuable agent, and that given in little larger doses than quinine, it is nearly or quite equal to the latter, with the advantage of producing less disturbance in the head.

